# Stimuli-Responsive Polysaccharide Hydrogels and Their Composites for Wound Healing Applications

**DOI:** 10.3390/polym15040986

**Published:** 2023-02-16

**Authors:** Maria Psarrou, Anna Mitraki, Maria Vamvakaki, Chrysoula Kokotidou

**Affiliations:** 1 Department of Materials Science and Technology, University of Crete, 70013 Heraklion, Greece; 2 Institute of Electronic Structure and Laser (IESL), FORTH, 70013 Heraklion, Greece

**Keywords:** polysaccharides, composites, stimuli-responsive, wound healing, biopolymers, hydrogels

## Abstract

There is a growing concern about wound care, since traditional dressings such as bandages and sutures can no longer meet existing needs. To address the demanding requirements, naturally occurring polymers have been extensively exploited for use in modern wound management. Polysaccharides, being the most abundant biopolymers, have some distinct characteristics, including biocompatibility and biodegradability, which render them ideal candidates for wound healing applications. Combining them with inorganic and organic moieties can produce effective multifunctional composites with the desired mechanical properties, high wound healing efficiencies and excellent antibacterial behavior. Recent research endeavors focus on the development of stimuli-responsive polysaccharide composites for biomedical applications. Polysaccharide composites, being sensitive to the local environment, such as changes of the solution temperature, pH, etc., can sense and react to the wound conditions, thus promoting an effective interaction with the wound. This review highlights the recent advances in stimuli-responsive polysaccharide hydrogels and their composites for use in wound healing applications. The synthetic approaches, physical, chemical, and biochemical properties as well as their function in wound healing will be discussed.

## 1. Introduction

A wound is defined as the disruption of the continuity of the epithelial lining of the skin. This is usually a result of an external trauma (physical injury, cutting of the tissue during surgery, thermal burn, etc.). Wound healing is a naturally occurring process, which comprises finely programmed phases involving hemostasis, inflammation, proliferation, and remodeling [1]. Wounds are classified as acute when they are a result of an accident or surgical injury and they have a typical healing rate of weeks, while chronic wounds include pressure ulcers, diabetic ulcers, and burns. Chronic wounds cannot be repaired in an orderly and timely manner and they fail to progress through the normal healing stages. Bacterial infection is the leading cause of the healing process failure. When bacteria colonize the wound area, the healing process is delayed, the inflammation response is extended, and usually skin re-epithelization fails, leading to worsening of the wound damage.

An ideal and effective wound dressing should be biocompatible, elastic, maintain the moisture around the wound, provide efficient protection from infections, be durable to external stress and possess good mechanical properties. Besides the above, recent approaches focus on the development of wound dressings that have the ability to interact and respond to the wound environment in order to achieve the maximum wound healing effect.

The skin wound microenvironment comprises two discrete components, the internal and the external microenvironments, both of which play a key role in the wound healing process [2]. The first refers to the skin area that surrounds the wound, while the second is defined as the exterior surface in contact with the wound. Even though the environmental conditions, such as the pH, O_2_, and CO_2_ levels, temperature, pressure, and hydration of the internal and external microenvironments are significantly different, the two microenvironments are in continuous exchange and impact each other. The specific conditions of the wounded area have recently guided the design of a plethora of stimuli-responsive dressings, which can strongly promote an effective wound healing process.

When the skin is injured the wound healing process starts immediately and is organized into four overlapping stages: (1) hemostasis, (2) inflammation, (3) proliferation, and (4) maturation. Initially, the hemostasis phase, which is also referred to as exudative or coagulation phase, involves the prevention of blood loss. In order to prevent excessive blood loss, a hemostatic reflex, vasoconstriction, occurs to restrict the blood flow. Platelets stick together and in combination with collagen and fibrin fibers produced by thrombin a platelet clot is formed [3]. The formation of this blood clot keeps the blood cells and platelets trapped inside the wound area. The injectability property of some stimuli-responsive polysaccharide hydrogels allows them to reach and have complete contact with the wounded area of interest, a useful property for aiding in the regulation of bleeding. The injectability and self-healing properties of polysaccharide hydrogels, in combination with their stimuli-response activity and subsequent drug release, can effectively accelerate the stages of wound healing by offering wound insulation and targeted delivery of antimicrobial or angiogenetic drugs [4].

The second stage of wound healing is the inflammation phase, also referred to as the defense mechanism of the organism. This phase focuses on the prevention of infection and removal of debris in order to prepare the area for the growth of new tissue. White blood cells called neutrophils enter the area for bacterial elimination followed by macrophages that clear the debris through phagocytosis and secrete proteins and growth factors that facilitate tissue repair. This happens about 48–72 h post-injury. In this phase, a localized swelling occurs due to the accumulation of white blood cells, enzymes, nutrients, and growth factors. Heat is also generated at this stage to combat infection [5].

The normal skin is naturally slightly acidic (pH 4–6) and acts as a barrier protecting the organism from bacteria and fungi. When the skin barrier is destroyed, the pH of the wound area first becomes alkaline (pH 7.5–8.9) and then gradually shifts to an acidic level during the wound healing process [2]. The local acidification is induced by the presence of bacteria strains in the infected wound area, which produce lactic or acetic acid. Moreover, reactive oxygen species (ROS) production, which results in an imbalance of the redox potential in the cells, is also increased in an infected chronic wound, due to the reduced concentration of antioxidants such as glutathione (GSH) [6,7]. In this regard, stimuli-responsive hydrogel dressings, which can release active biomolecules on demand to restore the local conditions in the wound area, have appeared as a very promising strategy for the treatment of chronic and infected wounds.

pH- and ROS- and enzyme-stimuli responsive polysaccharide-composite dressings are excellent contributors in facilitating this phase. Bacterial-infected areas tend to exhibit acidic pH values and increased ROS concentrations. Polysaccharide dressings that respond to these types of environments, either by releasing a drug when triggered to do so, or by partial degradation of the polysaccharide hydrogel and subsequent release of the drug, can enhance the normal bactericidal action, regulate inflammation and have a positive outcome in the wound healing procedure. Hydrogel composites that have hyaluronic acid as their main polysaccharide have the ability to degrade and release the encapsulated drug when exposed to bacterial infected areas. This degradation is the result of the hyaluronidase enzyme secreted by bacteria.

After the successful immune response and autolytic debridement, the wound healing moves to its third stage, the proliferation phase. During this phase, the wound contracts as new tissue made up of collagen and extracellular matrix is built. Fibroblast cells are essential at these stage since they comprise the structural framework of tissues and synthesize the extracellular matrix, a supportive framework for epithelial cells [8]. The wound is then covered by proliferating epithelial cells that create a layer acting as a protective barrier. In addition, angiogenesis is also present in the damaged area and a new network of microvascular blood capillaries are formed so that the granulation tissue receives sufficient nutrients and oxygen. Hydrogels composed of thermo-stimuli-responsive polysaccharide composites can be effectively used at this stage. The sol-gel transition of the hydrogel can be tuned to allow the release of growth factors that accelerate epithelial cell proliferation while simultaneously providing an adequate scaffold for the formation of the tissue matrix. Angiogenesis and collagen deposition can also be accelerated when the corresponding drugs are loaded into a stimuli-responsive polysaccharide hydrogel.

The last stage of wound healing is the maturation or remodeling phase. At this stage, the wound fully closes. Collagen deposited on the wound during the proliferation stage is remodeled from type III to type I. Then it is further cross-linked and aligned along tension lines with the aid of water reabsorption. Moreover, cells used during the previous stages that are no longer needed are removed by apoptosis or programmed cell death. The remodeling stage is initiated usually after 21 days post-injury and continues for more than 1 year. The healed areas do not immediately acquire their mechanical properties and tend to be weaker, having in general only 80% of the tensile strength of the uninjured skin.

Polysaccharides, which are the most abundant biopolymers found in nature, have attracted considerable attention as promising candidates for the development of next-generation “smart” biomaterials for application in various fields including drug delivery and wound dressings technology [9]. The unique properties of these sugar-based biopolymers include their high biocompatibility, biodegradability, absorption capacity, as well as the presentation of multiple functional sites of high chemical reactivity, which allow their facile derivatization and functionalization [10,11,12,13]. For example, polysaccharide materials have the ability to create and maintain a moist environment, while simultaneously keeping their absorption ability. Moreover, polysaccharide hydrogels possess a soft texture and stretching properties, an ideal combination for application as dressings on wounds located on the movable and stretchable parts of the body. Polysaccharides, including chitin, chitosan, dextran, collagen, cellulose, hyaluronic acid, and agarose have been extensively used for the development of hydrogel-based wound dressings [13]. However, the majority of natural polysaccharides lacks the responsiveness to environmental conditions or externally applied stimuli. In Table 1 are presented the main advantages and disadvantages of the most commonly used natural polymers in wound healing applications.

Stimuli-responsive polysaccharide-based hydrogels have gained increasing interest because of their ability to remotely change their physicochemical properties (e.g., solubility, shape) in response to an applied physical, chemical, or biochemical stimulus such as light, electric field, ultrasound, pH, temperature, ROS, and enzymes [14,15]. Compared to the traditional hydrogels used widely nowadays in wound healing, stimuli-responsive hydrogels may enable the spatially controlled release of bioactive molecules, such as anti-inflammatory agents, epidermal growth factors, hemostatic agents, and antimicrobial substances, at the wound sites, resulting in a faster healing process [16]. Moreover, depending on their components and synthetic pathways, such hydrogels can possess self-healing properties, which pose an advantage over traditional wound dressings and suture methods, since they allow the hydrogel dressings to maintain their efficacy and integrity when undergoing external forces during daily life, thus reducing the healing time and avoiding secondary injuries [17]. Finally, the injectability of certain wound healing polysaccharide hydrogels allows them to reach more complex wound areas, preventing the chances of bacteria invasion and infection. Research efforts have focused on the synthesis and characterization of stimuli-responsive polysaccharide-based hydrogels using robust synthetic routes (i.e., chemical modification reactions and crosslinking processes involving “click” chemistries and orthogonal synthetic strategies) aiming to develop multifunctional materials for use in wound healing applications [18].

**Scheme 1:**
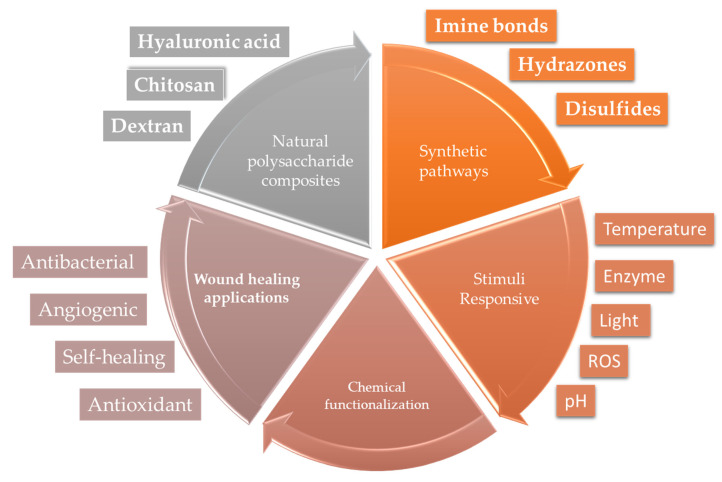
Flow diagram of the main objectives presented in this review.

In this review we will highlight the recent advances in stimuli-responsive hydrogels and their composite materials in wound management. In the first part of the article, we will summarize the synthetic methodologies and crosslinking processes employed for the development of “smart” polysaccharide hydrogels and composites, while the second part will exemplify the use of the different types of these smart materials in wound dressings/healing applications (Scheme 1). Emerging developments, challenges, and future trends in this exciting field are also discussed.

## 2. Chemical Functionalization of Polysaccharides

### 2.1. Oxidation of Polysaccharides

**Figure 1:**
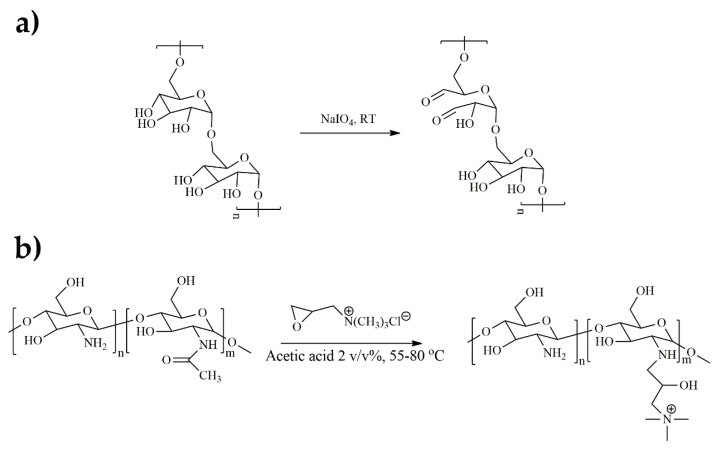
(**a**) Oxidation reaction of dextran using sodium periodate and (**b**) quaternization of chitosan by GTMAC (N-substitution).

The majority of polysaccharides (e.g., dextran, collagen, hyaluronic acid, etc.) comprise abundant hydroxyl groups in their chemical structure, which can be easily transformed into dialdehyde functional groups by a simple oxidation process using appropriate oxidizing agents such as sodium or potassium periodate. The oxidation of dextran is among the most widely studied processes (Figure 1a), while a similar synthetic procedure has been employed in the oxidation of other natural polymers; for example, sodium alginate, hyaluronic acid, and starch [19,20,21], as well as for hydroxyethyl cellulose [22,23] gellan gum [24,25], and β-cyclodextrin (β-CD) [26,27].

### 2.2. Modification of Chitin and Chitosan

Chitosan (CS) is a linear polymer derived from the deacetylation of chitin, a naturally occurring biopolymer that exists in the exoskeleton of crustaceans, insect cuticles, and fungi cell walls. CS is composed of randomly distributed units, namely: (1→4)-2-acetamido-2-deoxy-β-D-glucan (N-acetyl-D-glucosamine) and (1→4)-2-amino- 2-deoxy-β-D-glucan (D-glucosamine), linked by β(1→4) linkages, and possesses excellent biocompatibility, notable antimicrobial/antibacterial and hemostatic behavior as well as significant muco-adhesive properties [11].

Given its unique properties, CS has gained increasing interest as a promising candidate for use in various applications including wound healing. However, the solubility of CS under physiological conditions (pH 7.4) is limited due to the strong intra and intermolecular hydrogen bonds formed between its glucosamine units, which limit its further utility. To overcome the low biopolymer solubility, many studies have focused on the chemical modification of CS, via its structural amino and hydroxyl functional groups, to generate CS derivatives with improved properties, and flexibility in formulation development. Water-soluble CS derivatives prepared to date include quaternized CS (QCS), carboxymethyl CS (CMCS), and hydroxybutyl CS (HBCS) [20,28,29,30,31].

#### 2.2.1. Quaternization of Chitosan

QCS is a cationic ammonium salt derivative of CS, which has attracted great interest because of its enhanced antimicrobial activity and good water solubility. CS is transformed into QCS through a process called quaternization. There are several reports on different quaternization methods of CS; however the quaternization reaction using a cationic epoxide, glycidyl trimethyl ammonium chloride (GTMAC), has been extensively studied due to the facile synthesis and purification steps of the product as well as the high biocompatibility of the modified polymer (Figure 1b) [32,33,34]. In an aqueous solution containing 2 *v*/*v*% acetic acid and temperature ranging from 55 to 80 °C, CS is reacted with the epoxide group of glycidyl trimethyl ammonium chloride to give the water-soluble, cationic CS derivative. The reaction can take place either at the hydroxyl groups (O-substitution) or the amino groups (N-substitution) of CS (Figure 1b), whereas protection of the amino groups with a suitable protecting moiety, such as benzaldehyde, followed by the subsequent quaternization, results in the synthesis of solely O-substituted QCS [20].

#### 2.2.2. Carboxyalkylation of Chitosan

Another chemical reaction that is often used to increase the solubility of chitosan in water, and simultaneously provides amphoteric properties to the biopolymer, is carboxyalkylation. In this process, acidic groups are introduced, usually at the amino groups of CS, resulting in a polyelectrolyte containing both cationic and anionic functionalities. However, depending on the chemical process followed, the final carboxylated chitosan can be either O- or N- or N-, O-substituted [35]. Chitosan derivatives that can be obtained from a carboxyalkylation reaction are carboxyethyl, carboxymethyl, and carboxybutyl chitosan [30,36,37,38,39,40].

#### 2.2.3. Etherification of Chitosan

**Figure 2:**
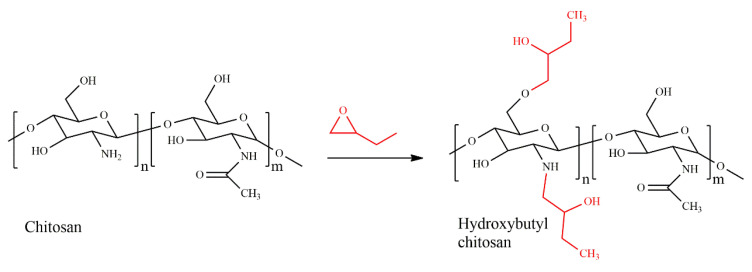
Synthesis of hydroxybutyl chitosan.

HBCS is a water-soluble CS derivative, synthesized by the conjugation of hydroxybutyl groups to the hydroxy and amino groups present on the CS backbone, and exhibits a lower critical solution temperature (LCST), which is dependent on the degree of hydroxybutylation (Figure 2) [29]. HBCS is biocompatible and biodegradable, while it also possesses antibacterial properties and provides a moist environment, which are ideal for use in wound healing applications [41].

HBCS is obtained by two different synthetic approaches: homogeneous synthesis and the heterogeneous synthesis [29,42,43]. The first method involves the dispersion of CS in an alkaline urea solution followed by freeze-thawing and subsequent reaction with 1,2-butene oxide at ambient temperature. In the second method, CS is first dispersed in a strong alkaline solution (alkalization) and next an organic dispersant is added, followed by 1,2-butene oxide, which is grafted onto the CS chains at elevated temperatures between 55 and 60 °C. Due to the strong alkaline conditions and the high temperatures used in the heterogeneous synthesis, polymer degradation can occur, and therefore, the homogeneous method is preferred. Following a similar synthetic procedure, thermoresponsive hydroxypropyl chitin has been prepared [44,45,46].

## 3. Stimuli-Responsive Polysaccharide-Based Hydrogels

### 3.1. pH-Responsive Polysaccharide Hydrogels

**Scheme 2:**
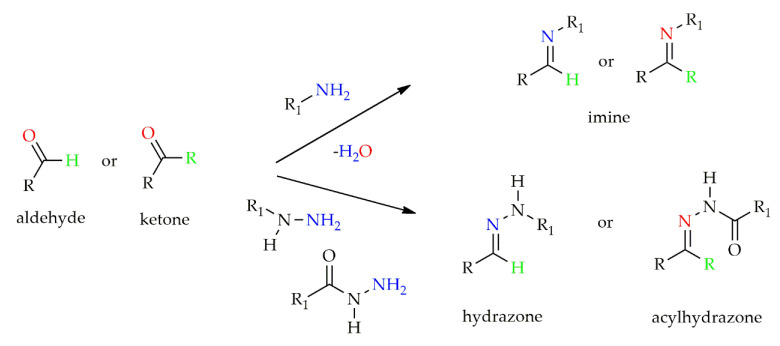
Formation of Schiff base linkages.

Stimuli-responsive hydrogel dressings presenting labile bonds within the hydrogel matrix have attracted great attention in wound healing applications. Imine, hydrazine, and acylhydrazone bonds, commonly known as Schiff bases, have been extensively used for the synthesis of pH-sensitive wound dressings. These bonds are easily formed via the reaction of a carbonyl group, an aldehyde, or a ketone, with a primary amine, a hydrazine or a hydrazide, respectively (Scheme 2), and possess reversible properties, thus enabling the formation of self-healing materials. Moreover, it was shown that hydrogels based on reversible Schiff base junctions possess higher mechanical strength compared to their physically cross-linked counterparts [32].

Polysaccharides bearing aldehyde functionalities are commonly derived from the oxidation of the natural polymers, and have been widely employed in the synthesis of hydrogels bearing acid-sensitive Schiff base cross-links. Such polymers include oxidized hyaluronic acid (OHA) cross-linked with carboxymethyl chitosan to obtain a hydrogel for diabetic wound dressing [47]. Alternatively, aldehyde functionalized synthetic polymers of complex architecture, 4-arm poly(ethylene glycol) (PEG), have been also cross-linked with natural polysaccharides such as carboxymethyl chitosan for use in chronic wound healing [30].

pH-responsive nanocomposite hydrogels comprising chitosan, oxidized hydroxypropyl cellulose (HPC), and a water-soluble POSS, octa (γ-chloroammoniumpropyl) silsesquioxane (OCAPS), were also reported by Zhang et al. [48]. The acid-labile Schiff base linkages were formed by the reaction of the aldehyde groups of HPC with the amine groups of chitosan, while OCAPS was bound via imine bonds onto HPC. In another example, pH-responsive multi-composite hydrogels based on the natural polymers arabinoxylan and carrageenan, combined with reduced graphene oxide (rGO) and cross-linked with tetraethyl orthosilicate (TEOS) through hydrogen bond formation, were developed by Khan et al. [49]. The hydrogels exhibited a pH-dependent swelling, with maximum degree of swelling at neutral pH 7, and a decrease in their swelling under both acidic and alkaline conditions. Moreover, as it was found from the stress–strain curves, the composite hydrogel exhibited higher Young’s modulus compared to the natural human skin while the tensile strength was increased by increasing the rGO concentration in the material. As a result, the final composite material had better mechanical properties than the natural human skin.

**Figure 3:**
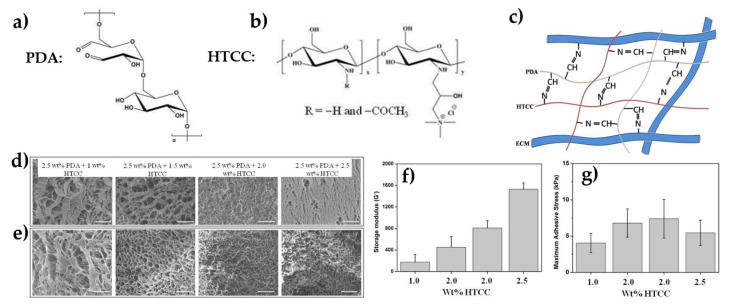
Chemical structure of oxidized dextran (**a**), the cationic chitosan derivative (**b**) and the OD-HTCC hydrogel (**c**). Surface (**d**) and cross-section (**e**) SEM images of the hydrogels at different HTCC concentrations. Scale bar 50 μm. Storage modulus of the hydrogel as a function of HTCC concentration (**f**) and adhesive strength measured using porcine skin (**g**). Adapted with permission from [50].

Functional wound dressings that combine a pH-responsive behavior with effective antibacterial moieties, have recently emerged as an attractive approach to control the inflammation response in the wound area and speed up the wound healing process. In 2017, Hoque and coworkers presented the in situ formation of an antibacterial and bioadhesive hydrogel, comprising oxidized dextran (OD) and cationic quaternized chitosan [50]. OD, at a degree of oxidation of 51 ± 1%, was synthesized by the reaction of the hydroxyl groups of dextran with sodium periodate, whereas CS was quaternized using GTMAC to produce the antibacterial polymer N-(2-hydroxypropyl)-3-trimethylammonium chitosan chloride (HTCC) (44% degree of quaternization) (Figure 3a,b). OD-HTCC hydrogels were prepared by the reaction of the aldehyde groups of OD with the free amino groups of HTCC to confer acid-labile Schiff base cross-links. The hydrogels were formed within 10–60 sec (Figure 3c), depending on the HTCC concentration (2, 3, 4, and 5 *w*/*w*%), with the lower polymer concentrations leading to faster gelation times, highly porous structures (Figure 3d,e) and a gradual decrease of the storage modulus (G’) (Figure 2f). Moreover, the hydrogels presented high adhesive stresses in the range of 4.05 to 7.4 kPa (Figure 3g), showing good bioadhesion, which is usually a limitation of most of the traditional bioadhesives.

In related studies, OHA was employed to prepare acid-sensitive, antimicrobial hydrogels via the cross-linking of its aldehyde groups using adipic acid dihydrazide modified hyaluronic acid (HA-ADH) to form acylhydrazone junctions. Sisomicin sulfate or N(O)-protected QCS were chemically bound onto the aldehyde groups of the hydrogels via imine linkages to confer the antimicrobial properties to the hydrogels [20,51].

More recently, composite hydrogels comprising QCS cross-linked with OHA through imine bond formation were prepared [32]. The hydrogels were loaded with a natural antibacterial agent, berberine (BBH), the epidermal growth factor (EGF), and poly(3, 4-ethylenedioxythiophene):poly(styrene sulfonate) (PEDOT:PSS) as a photothermal agent, and released their cargo at the acidic wound area. The presence of PEDOT allowed maximization of the antibacterial effect in a controllable manner, upon near infrared (NIR) light irradiation.

Of great interest in the development of “smart” wound dressings are also hydrogels combining pH-responsive properties with a self-healing action that maintains the integrity of the dressing when applied to deformable parts of the body. pH-responsive, self-healable hydrogels based on QCS and aldehyde functionalized Pluronic F127 (PF127-CHO) micelles for joint skin wound dressings were prepared [52]. QCS was synthesized using GTMAC, while PF127-CHO was prepared by the reaction of PF127 with 4-hydroxybenzaldehyde following the mesylation of the hydroxyl groups of PF127. The PF127-CHO curcumin-loaded spherical micelles, decorated with functional aldehyde groups at the outer shell, were reacted with the amine groups of QCS to obtain the hydrogels via imine bond formation. The excellent self-healing ability of the hydrogels was attributed to the Pluronic micelle physical cross-linking interactions and the Schiff-base bonds between the micelles and the QCS.

**Figure 4:**
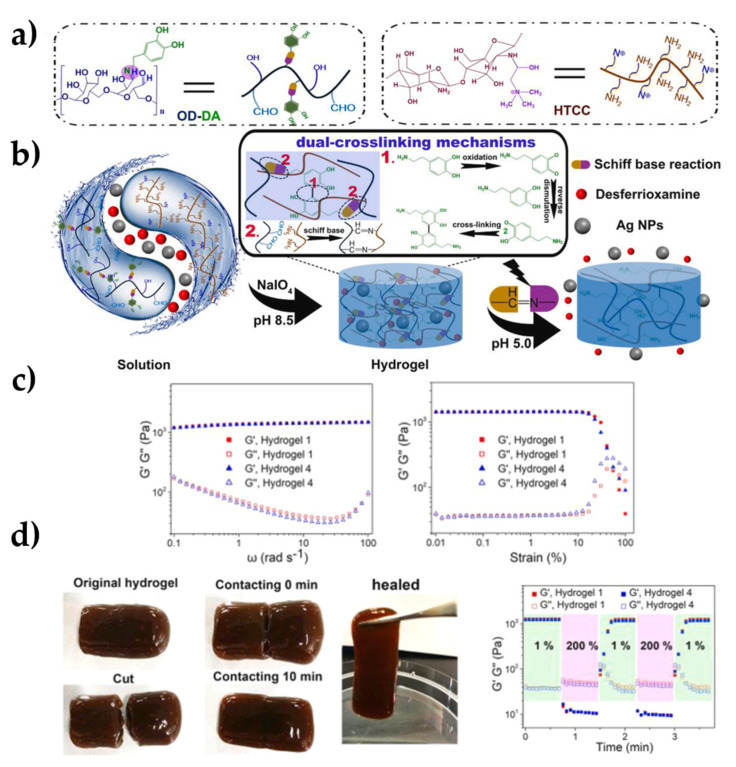
Chemical structures of OD–DA and HTCC: (**a**) schematic representation of the dual-cross–linking mechanism and the cleavage of the Schiff base bonds, followed by the release of DFO and AgNPs; (**b**) G’ and G’’ of the hydrogels as a function of the frequency and the % applied strain; (**c**) self-healing behavior of hydrogel 4 and alternate step strain sweep experiments for hydrogel 1 and hydrogel 4 at strains ranging from 1 to 200% (**d**) Note: Hydrogel 1: control hydrogel, Hydrogel 2: hydrogel@AgNPs, Hydrogel 3: hydrogel@DFO, Hydrogel 4: hydrogel@AgNPs-DFO. Adapted with permission from [53].

In another study, mussel-inspired, pH-responsive, dual-cross-linked, injectable, and adhesive hydrogels for the treatment of chronic diabetic wounds were developed [53]. The “smart” hydrogels were formed by the reaction of dopamine-conjugated oxidized dextran (OD-DA) with the antibacterial polymer HTCC, via the formation of acid-labile imine bonds between the aldehyde groups of dextran and the free amine groups of HTCC (Figure 4a). After gelation, dopamine was oxidized using sodium periodate to form catechol–catechol junctions within the hydrogel matrix, which led to a double cross-linked network (Figure 4b). The incorporation of silver nanoparticles (AgNPs) and the pro-angiogenic drug deferoxamine (DFO) within the pores of the hydrogel enhanced its antibacterial properties and conferred an angiogenic character, without affecting the viscoelastic properties of the hydrogels (Figure 4c). Under acidic conditions, such as those found in an infected diabetic wound (pH 4–6), the Schiff base bonds were cleaved, resulting in the release of the AgNPs and DFO at the wound site. Moreover, the hydrogels were tested for their self-healing properties. Complete gel degradation was found under an applied stress of 200%, leading to G’’ > G’ and a transition to the liquid state, while the hydrogel was recovered and its mechanical properties were restored when releasing the stress, indicating the dynamic nature of the hydrogels (Figure 4d).

**Figure 5:**
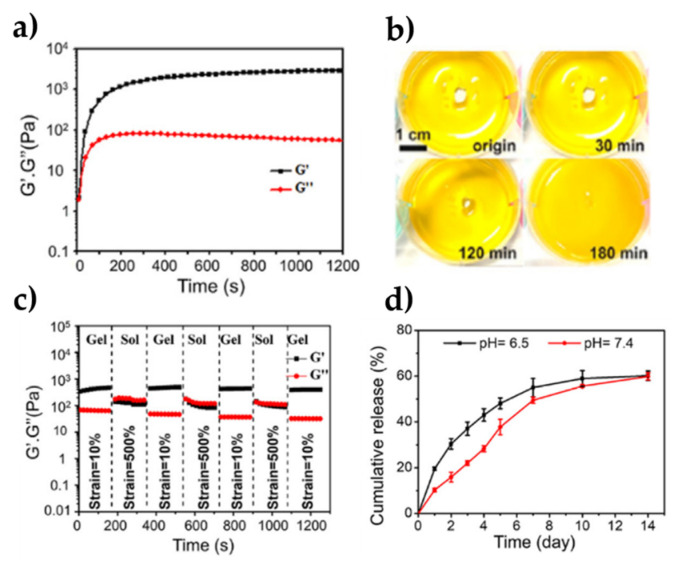
(**a**) G’ and G’’ of the hydrogel as a function of time, (**b**) optical observation of the self-healing process; (**c**) G’ and G’’ of the hydrogel as a function of time during several sol-gel transition cycles; and (**d**) release profiles of insulin from the hydrogel at pH 7.4 and 6.5. Adapted with permission from [38].

Finally, Li and coworkers, presented a pH-responsive, self-healing hydrogel, based on N-carboxyethyl chitosan (N-chitosan) and OHA cross-linked with adipic acid dihydrazide (ADH) via imine and acylhydrazone bond formation, respectively, for diabetic wound healing [38]. N-chitosan was synthesized by the reaction of chitosan with acrylic acid under alkaline conditions, while OHA was obtained by oxidation of HA with sodium periodate. Stable hydrogels were formed within 30 sec following the mixing of dilute solutions of OHA, N-CECS, and ADH (Figure 5a). The self-healing properties of the hydrogels were evidenced visually by the gradual disappearance of a hole at the center of the hydrogel after 3 h (Figure 5b), as well as by measuring the storage and loss moduli (G’, G’’) of the hydrogels during several sol-to-gel cycles (Figure 5c). Insulin was loaded within the porous of the hydrogels to enhance the wound healing process by promoting the re-epithelization of the damaged skin and improving angiogenesis. The release of insulin was faster at pH 6.5 compared to neutral conditions due to the cleavage of the acid-labile acylhydrazone and imine bonds (Figure 5d) [38].

### 3.2. Redox-Responsive Polysaccharide Hydrogels

The redox potential in a damaged and infected skin area changes during the healing process, and therefore dressings which respond to a redox stimulus are highly desirable. Disulfide bonds are known to undergo reversible formation upon oxidative and reductive conditions and have been commonly used in the fabrication of redox-responsive materials. Gao et al. proposed a redox-responsive hydrogel comprising HA cross-linked with a GSH-sensitive cross-linker, aminoethyl disulfide (AED), via the formation of amide bonds between the amino groups of AED and the carboxylic acid groups of HA [7]. The prepared hydrogel was biocompatible and aided fibroblast growth and proliferation over seven days, whereas its disulfide bonds at the cross-link junctions were cleaved in the presence of GSH, resulting in the gradual disintegration of the hydrogel. Given the lower GSH levels in diabetic foot ulcers compared to those of the healthy tissues, the morphological changes of the GSH-sensitive hydrogels were proposed for use in identifying the healthy tissue during the healing process.

### 3.3. Photo-Responsive Polysaccharide Hydrogels

**Figure 6:**
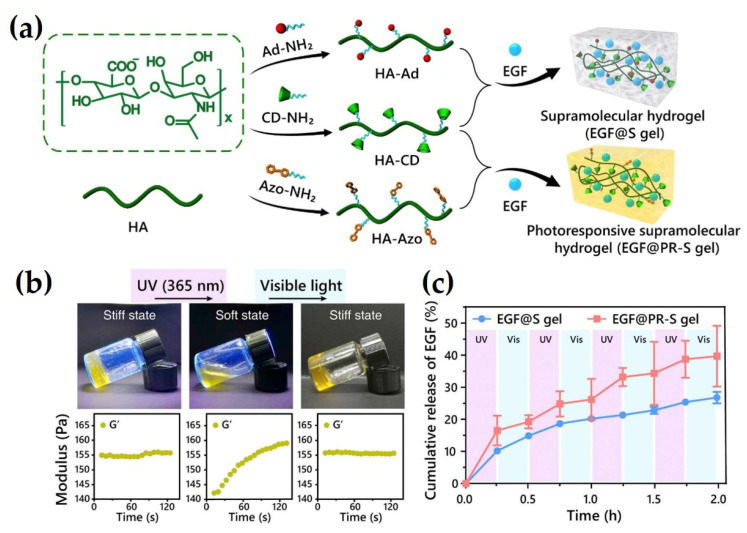
Schematic illustration of the synthetic procedure followed for the preparation of the photo-responsive hydrogel: (**a**) photographs showing the sol-gel transition and the corresponding moduli following irradiation with UV and visible light; and (**b**) the release profile of EGF from the hydrogels upon alternating UV and visible light irradiation (**c**). Adapted with permission from [54].

Photo-responsive hydrogels are highly attractive in wound healing, allowing the spatiotemporal control of the applied stimulus, and thus of the mechanical properties and the release profiles of encapsulated actives from the hydrogels. Zhao and coworkers synthesized a novel, light-responsive supramolecular hydrogel based on host–guest interactions between azobenzene and β-cyclodextrin (β-CD) moieties grafted along hyaluronic acid chains [54]. Azobenzene is a well-known photo-responsive molecule that undergoes a trans-to-cis transition upon UV light irradiation. Trans-azobenzene has a high affinity for the hydrophobic cavity of β-cyclodextrin, thus promoting the formation of host–guest interactions, whereas cis-azobenzene does not interact with β-cyclodextrin. HA chains conjugated with cyclodextrin (HA-CD) were spontaneously interacted with HA chains conjugated with trans-azobenzene (HA-Azo) to form a stiff supramolecular hydrogel with G’ ~155 Pa (Figure 6a). Upon UV irradiation, G’ decreased to ~144 Pa, indicating the reduction of the cross-link density of the hydrogel due to the isomerization of azobenzene from the trans to the cis isomer, whereas G’ was gradually recovered in the dark or under visible light irradiation, which promote the back isomerization of azobenzene to the trans conformation. Finally, encapsulation of EGF within the hydrogel cavities enabled the photo-controlled release profile of the growth factor, which promoted the wound healing and angiogenesis processes (Figure 6c).

### 3.4. Dual Responsive Hydrogels

#### 3.4.1. pH- and Temperature-Responsive Polysaccharide Hydrogels

Thermoresponsive polymers exhibiting a critical solution temperature can form reversible gels above or below this temperature. In particular, polysaccharide-based polymers with a lower critical solution temperature close to the body temperature are very attractive for wound healing applications due to their in-situ gelling ability [55]. On the other hand, hydrogels with a dual responsive character, recognizing independently or synergistically two orthogonal stimuli, may exhibit a dynamic behavior that mimics the complexity of natural systems.

Wang and co-workers synthesized pH- and the thermoresponsive hydrogels with UV-shielding properties for diabetic wound healing [56]. The fabricated hydrogels consisted of the natural polymer pullulan oxidized to obtain aldehyde pullulan (APu) and cross-linked with Pluronic F127-grafted polyethylenimine (PEI) via Schiff base bond formation. The temperature-responsive gelation of the hydrogels was tested at 4, 25, and 37 °C for different APu concentrations and a sol-to-gel transition was observed at temperatures above 25 °C [56]. Adipose mesenchymal stem cell (ADSC)-derived exosomes were loaded within the hydrogel via electrostatic interactions. A more pronounced release of the exosomes from the hydrogel was observed at pH 5.5 upon cleavage of the acid-labile imine bonds, which promoted an effective wound healing process.

Another pH- and thermoresponsive, multi-composite hydrogel based on hydroxypropyl chitin (HPCH) conjugated with tannic acid (TA) and ferric ions was fabricated by Ma et al. [45]. HPCH was synthesized by grafting propylene oxide onto the chitin chains under alkaline conditions and the multi-composite HPCH/TA/Fe hydrogels were prepared by simply mixing the components at the desired concentrations. Mixtures of TA at concentrations above 4 mg/mL and HPCH at 2 wt% led to the formation of coacervates rather than a homogeneous network. It was also observed that for TA concentrations in the range between 0.5−3 mg/mL the hydrogels did not exhibit thermoresponsive properties. To overcome this limitation, the authors used Fe ions that coordinated with TA while retaining the reversible thermoresponsive behavior of the hydrogel. Oscillatory rheology measurements showed that for the HPCH/TA/Fe mixture, the sol-to-gel transition took place at ~18 °C and was complete at 37 °C. Moreover, the release profile of TA from the composite hydrogels at different pH values was investigated. An increase in the release was found under acidic conditions (pH 3.0), which decreased in neutral and alkaline conditions due to the deprotonation of the pyrogallol/catechol groups of TA, which complex strongly with the Fe ions [45].

#### 3.4.2. pH- and ROS-Responsive Polysaccharide Hydrogels

**Figure 7:**
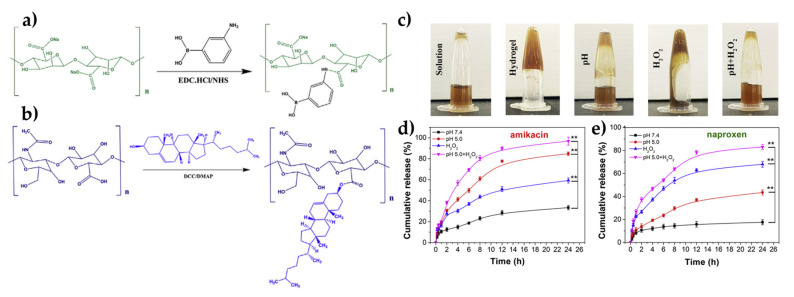
Schematic representation of the synthetic process followed for the synthesis of ALG-BA (**a**) and HA-CHOL (**b**). Photographs showing the formation of the hydrogel and its stimuli-responsive behavior (**c**) and percentage cumulative release of amikacin (**d**) and naproxen (**e**) from the hydrogels as a function of time for different incubation conditions (mean ± SD, n = 3), * *p* < 0.01, ** *p* < 0.001. Adapted with permission from [57].

Dual pH- and ROS-responsive hydrogels are compelling candidates for infected wound management, due to the acidic conditions and the high ROS concentration found in these wounds [6]. Recently, Hu et al. proposed a dual pH- and ROS-responsive hydrogel [57] based on sodium alginate modified with 3-aminophenyl boronic acid to attach the boronic acid functionalities to the alginate backbone (ALG-BA) (Figure 7a). HA was esterified with cholesterol (CHOL) molecules, resulting in an amphiphilic HA-CHOL polymer (Figure 7b). Under alkaline conditions (pH 8–9), ALG-BA formed a hydrogel bearing dynamic pH- and ROS-sensitive boronic ester bonds. The amphiphilic polymer HA-CHOL self-assembled into spherical nanoparticles that were loaded with the hydrophobic, anti-inflammatory drug naproxen. The drug-loaded HA-CHOL micelles were entrapped in the interior of the hydrogel matrix along with amikacin as an antibacterial drug. At acidic conditions and high ROS concentration, the hydrogel was dissociated due to the cleavage of the boronic ester bonds, resulting in the accelerated release of the anti-inflammatory and antibacterial drugs (Figure 7c,d) [57].

#### 3.4.3. pH- and Electro-Responsive Polysaccharide Hydrogels

**Figure 8:**
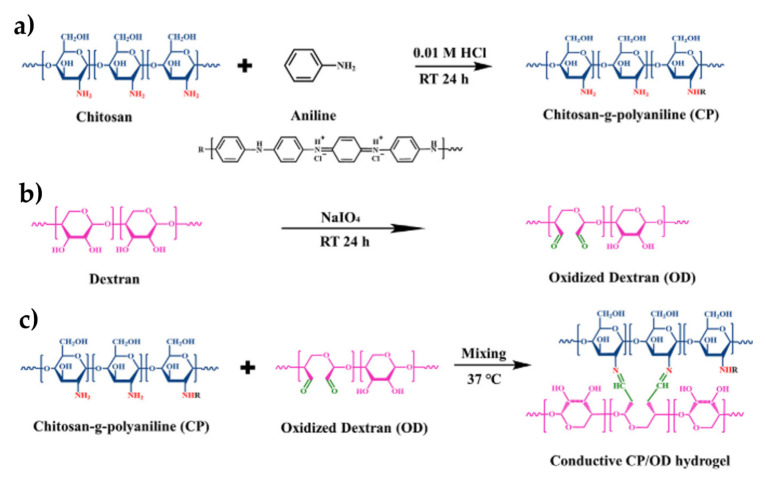
Synthesis of (**a**) chitosan-*g*-polyaniline (CP), (**b**) oxidized dextran (OD), and (**c**) the chitosan-*g*-polyaniline/oxidized dextran (CP/OD) hydrogel. Adapted with permission from [58].

Electrical stimuli have recently emerged as another attractive avenue to address chronic diseases that need frequent injections or precise doses of medicine. The facile and controlled application of an electrical field renders it a suitable tool for practical use in drug delivery systems and wireless implants. Conducting polymers are electro-responsive materials which, among other benefits, combine good biocompatibility and inherent antibacterial properties. In this context, Qu et al. developed a “smart” dual pH- and electro-responsive polysaccharide hydrogel [58] based on a chitosan-*g*-polyaniline (CP) graft copolymer (Figure 8a). The CP polymer was reacted with oxidized dextran (OD) (Figure 7b) to form a hydrogel bearing pH-labile imine bonds (Figure 8c), which presented impressive responsiveness both to solution pH variations and to an applied electric field. Encapsulation of the hydrophilic negatively charged drug amoxicillin, within the hydrogel showed a pH-dependent release profile, whereas application of an external electric field at an applied voltage between 0 and 3 V led to voltage-dependent release profiles of both hydrophilic and hydrophobic drugs. In a similar context, this group employed an oxidized hyaluronic acid-*graft*-aniline tetramer and N-carboxyethyl chitosan (N-CECS), cross-linked via Schiff base linkages to develop multi-functional wound dressings presenting a pH- and electro-responsive behavior, as well as higher granulation tissue thickness, collagen disposition, and angiogenesis for improved skin regeneration [39].

## 4. Polysaccharide-Peptide Composites with Cell Instructive/Responsive Properties

Although polysaccharide materials are excellent materials for wound healing, they lack the cell instructive/responsive properties that are conferred by cell-adhesive moieties. The most common cell-adhesive motif is the tripeptide RGD (arginine-glycine-aspartate) from fibronectin that attaches to integrin receptors. Conjugation of the RGD motif has been extensively reported mainly for peptide and protein scaffolds towards the design of functional biomaterials for tissue engineering [59,60]. A number of studies have reported the functionalization of polysaccharide-based materials with the RGD motif. The first chemical strategy used for grafting the peptide GRDGDY to alginate was via aqueous carbodiimide chemistry, resulting in the formation of an amide bond between the N-terminal amino group of the peptide and the carboxylate groups of alginate [61]. The same strategy can be used for other polysaccharide materials, for example, chitosan. A water-soluble derivative of chitosan, carboxymethyl-trimethyl chitosan (CM-TM-CS), grafted with GRGDS sequences using carbodiimide chemistry, displayed enhanced cell adhesion of human dermal fibroblasts, suggesting its potential applicability in wound healing [62]. Another strategy followed was the conjugation of maleimide-functionalized alginate capsules with thiol-terminated RGDS peptides (CRGDS) [63]. The presence of the peptides resulted in the acceleration of the proliferation of the encapsulated fibroblasts within the alginate capsules, suggesting their potential use in skin regeneration applications. Moreover, gelatin methacrylamide (GelMA) contains cell-binding motifs and adheres well to tissues. Alginate-GelMA composite hydrogels with encapsulated mesenchymal stem cells were reported to accelerate wound healing. [64]. Further peptides that can be grafted to alginate or other polysaccharide hydrogels include differentiation-inducing peptides, peptides sensitive to matrix metalloproteinases (MMPs), and antimicrobial peptides. Functionalization with MMP-sensitive sequences allows the cleavage of the hydrogels by MMPs and the remodeling of the extracellular matrix. Alginate scaffolds have been functionalized with both RGD and PVGLIG sequences, the latter being sensitive to cleavage by MMPs between the G and L residues [65]. The authors concluded that functionalization with both peptides improved the behavior of the alginate matrix as a dynamic ECM analog compared to the alginate matrix functionalized solely with RGD. Very recently, Suo et al. presented a hyaluronic acid-based composite hydrogel with a pH-responsive behavior. The proposed hydrogel comprised oxidized hyaluronic acid (OHA) cross-linked with the antimicrobial peptide KK(SLKL)_3_KK via the formation of imine bonds between the aldehyde groups of OHA and the free amine groups of the peptide [66]. Cleavage of the Schiff base bonds in acidic pH allowed the release of the antimicrobial peptide, and since the pH of bacteria-infected wounds is around 5.5, resulted in a full-spectrum antibacterial activity. Following a similar approach, Gao’s group reported on a dynamic hydrogel comprising oxidized dextran (OD) cross-linked with the antimicrobial peptide ε-poly-L-lysine (EPL) through Schiff base formation [67].

## 5. Applications in Wound Healing/Dressings

### 5.1. pH-Responsive Polysaccharide Hydrogels

As discussed above, the healing procedure is affected by the pH, which typically changes between pH 4 and 6 due to the increase of the population of common bacteria, such as *Escherichia coli* (*E. coli*) and *Staphylococcus aureus (S. aureus)*, and between pH 7 and 9 in basic milieu for both chronic and acute wounds. The pH oscillation is dependent on the type of bacteria and their toxicity, the type of tissue, the angiogenesis procedure, the presence of proteases, the oxygen release, and the phase of the healing process [68]. The aforementioned parameters should be taken into careful consideration when developing pH-responsive wound dressings. To measure these oscillations, a wound healing and pH-monitoring polysaccharide dressing was developed by combining 3 wt% chitosan with 1 wt% carbon dots to form a film through the solvent casting method. The nanocomposite film was proven to be pH-sensitive, exhibiting a color change from bright yellow to dark yellow when the pH value increased, and rendering the CDs visible under UV exposure or even under daylight, enabling the use of the film in monitoring the pH during the healing process. The high sensitivity of the carbon dots at lower pH values was attributed to their aggregation, which followed the protonation of the carboxylic acid groups on the surface of the CDs [69]. Furthermore, the film exhibited high biocompatibility when tested on the L929 fibroblast cell line, as well as excellent bactericidal activity against *S. aureus* by completely inhibiting the growth of the bacteria when the concentration of CDs was higher than 10 μg/mL. In vivo experiments were conducted on rats to evaluate the use of the CDs/chitosan nanocomposite film as an antibacterial wound healing bandage and showed that the healing process was noticeable after 9 days, leading to a complete wound closure after 18 days [70].

The pH oscillations offer a convenient stimulus for the pH-dependent release of bioactives, which promote the healing process at the wound site. A pH-responsive mussel-inspired hydrogel with controlled cargo release properties was developed by Hu et al. [53] for chronic infected diabetic wound treatment. The hydrogel comprised HTCC cross-linked with OD-DA, and incorporated silver nanoparticles and the pro-angiogenic drug deferoxamine, offering both bactericidal and angiogenic properties. When exposed to an acidic and bacteria-infected environment, the drug-loaded hydrogel released its cargo in a sustained manner. Angiogenesis and subsequent wound healing were accelerated by the death of the bacterial population and the increase in the expression of the hypoxia-inducible factor-1 alpha (HIF-1α) and vascular endothelial growth factor (VEGF).

**Figure 9:**
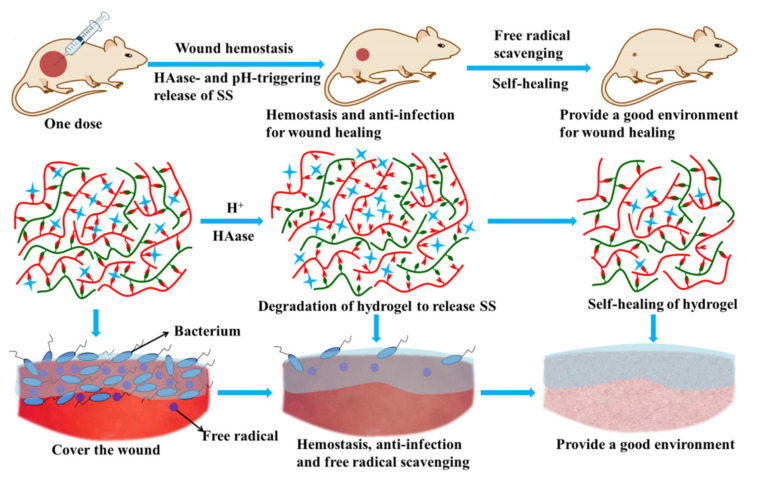
Schematic representation of the pH- and HAase-sensitive A-HA/HA-ADH/SS hydrogel functions, regarding hemostasis, antimicrobial action, free radical scavenging, and self-healing properties. Adapted with permission from [51].

Dual stimuli-responsive hydrogels trigger two or more functions simultaneously and thus offer an improved wound healing process. Such a “smart” pH- and hyaluronidase-sensitive hydrogel was developed by Guan et al. by cross-linking OHA with HA-ADH, for potential clinical use as an effective antibacterial dressing [51]. Hyaluronic acid, the main component of the hydrogel, exhibited a good water-holding capacity and offered the advantage of being degraded within the infected wound area, due to the bacteria-secreted HAase enzyme [71,72]. This allowed the release of the aminoglyglycoside antibiotic sisomicin sulfate (SS), which was loaded onto the network via dynamic imine bonds. The hydrogel showed a fast degradation rate in acidic and HAase-rich conditions, on-demand release of SS, and a sustained antibacterial effect against *S. aureus* and *E. coli* (Figure 9). Histological examination on a full-thickness mouse skin defect model exhibited reduction of the inflammation reaction and in the population of the pathogens, in addition to a high healing ratio by reducing the wound area by 46% within 10 days. Moreover, the hydrogel was able to self-heal when an external force was applied by reconstituting its imine and acylhydrazone bonds.

A triple functional, pH-responsive, ultra-stretchable, and antibacterial composite hydrogel was developed using a poly(vinyl alcohol)-borax (PB) gel as the matrix, dually reinforced with dopamine-grafted oxidized carboxymethyl cellulose (OCMC-DA) and cellulose nanofibers [73]. Neomycin, a model antibacterial drug, was covalently cross-linked via its multiple amino groups onto the OCMC-DA polymer network via Schiff base linkages. The cross-linking of all the components conferred to the fabricated hydrogel excellent mechanical properties and stretchability that reached 3300%, allowing its application on wound areas in which high motion is experienced, such as the skin around joints. The hydrogel exhibited a pH-triggered degradability accompanied by the controlled release of neomycin in vitro at both pH 5 and pH 7.4, reaching a 40–47% release after 1 day, due to the degradation of the imine linkages between the amino groups of neomycin and the aldehyde groups of OCMC-DA.

### 5.2. Thermoresponsive Polysaccharide Hydrogels

Temperature is another convenient stimulus allowing tuning of the sol-gel transition as well as the release profile of encapsulated bioactive molecules. A thermosensitive hydrogel that can release the encapsulated keratinocyte growth factor (KGF) and enhance the wound healing of endometrial injury was developed by Xu et al. [74]. The mucoadhesive hydrogel comprised a heparin-modified poloxamer (HP) cross-linked with ε- polylysine (EPL). HP served as the matrix material and exhibited a good gelation profile, as well as strong affinity and stabilizing properties for some growth factors [75]. EPL acted as a multifunctional excipient since it rendered the hydrogel bioadhesive and efficiently increased the retention time of the released KGF in the endometrial mucosa, which resulted in more KGF being absorbed by the injured uterus. KGF is a regulator of the migration and proliferation of epithelial cells and interactions [76]. Endometrial wounds suffer from poor retention and low absorption efficiency of therapeutic drugs due to endometrial mucus, which acts as a biological barrier [77]. By adjusting the EPL content (90 ug/mL) in the hydrogel formulation, the adhesion force of the hydrogel was increased up to 3.18 N, and the mechanical properties and KFG release were greatly enhanced. As a result, the KGF-EPL-HP-90 hydrogel enabled the repair of endometrium in the injured uterus within 3 days in an IUA rat model, and enhanced the proliferation and keratinization of the epithelial cells due to the accelerated release of KGF.

A dual pH- and temperature-responsive injectable hydrogel exhibiting antibacterial and wound healing properties is described in the work of Ma and coworkers [45].

The hybrid gel composite consisted of the polysaccharide HPCH, which provided the moist environment required for tissue regeneration, tannic acid (TA), which acted as the antimicrobial agent [78], and Fe^3+^ ions, which served as the cross-linker for HPCH [79] and enhanced the mechanical properties of the hydrogel. The pre-cooled HPCH/TA/Fe solution at low temperatures was injected into the infected wound area, where it gelled very rapidly due to the increase of the temperature to 37 °C. On the other hand, an acidic infected wound triggered the release of tannic acid from the hydrogel. In vitro studies using NIH3T3 mouse skin fibroblast cells showed a migration-promoting stimulation and negligible cytotoxicity, verifying the biocompatibility of the hydrogel. In vivo experiments on a *S. aureus*-infected full thickness cutaneous mouse model showed that the application of the HPCH/TA/Fe hydrogel accelerated the wound repair by presenting a long-lasting antimicrobial activity for up to 7 days and resulted in scar-free healing.

One of the major challenges nowadays in wound management is diabetic wound healing and angiogenesis. An injectable, multifunctional dressing was developed by Wang et al. [56] as a scaffold for the pH-responsive release of exosomes, aiming to enhance diabetic wound healing. Polysaccharide-based fluorinated ethylene propylene (FEP) [80] dressings offer multiple advantages including efficient antibacterial activity, UV shielding, self-healing ability, and fast hemostatic activity. Adipose-derived MSC (ADSC) exosomes are gaining increased attention for use in wound healing applications, due to their stability and nanometer size and their function in fibroblast regulation [81,82]. In addition, there is a low chance of triggering an immune response. However, the use of exosomes as therapeutic agents faces many challenges due to their rapid clearance and short half-life when administered in vivo. To address these challenges, the ADSC-derived exosomes were loaded in a FEP scaffold via electrostatic interactions. Application of the FEP@exo scaffolds in vivo exhibited excellent retention of the bioactivity of the exosomes and a sustained long-term release, resulting in the promotion of the angiogenic process, collagen deposition, re-epithelialization, and a faster diabetic wound healing process.

### 5.3. Enzyme-Responsive Polysaccharide Hydrogels

As discussed above, bacterial-induced inflammation is a primary concern in wound healing as well as in tissue engineering. To address this issue, hydrogels have been loaded with antibacterial agents, such as antibiotics or silver nanoparticles, either via physical loading, or via chemical interactions [83,84]. This method, although beneficial, suffers from a short window of effective action, bacterial resistance, and potential toxicity towards mammalians cells. Multiple studies have been conducted aiming to develop a hydrogel that can offer on-demand antibiosis, and simultaneously present self-healing properties to avoid rupture and loss of function caused by external forces during the daily life usage. In a novel study, Tian et al. [85] developed a hyaluronic acid (HA)-based hydrogel with germ-triggered antimicrobial properties. HA was cross-linked via ethylenediaminetetraacetic acid (EDTA)−Fe^3+^ complexes. Release of Fe^3+^ was triggered in the presence of bacteria that excreted the HAase enzyme in the wound-infected area. The HAase enzyme breaks down hyaluronic acid, thus enabling the release of the complexed Fe^3+^ ions, which were rapidly adsorbed by the surrounding bacteria. Next, Fe^3+^ was reduced to Fe^2+^, which reacted with H_2_O_2_ and formed a hydroxyl radical that damaged the bacterial cell and led to bacterial death. The prolonged release of the antibacterial agent Fe^3+^ sustained the antibacterial action of the hydrogel when a bacterial infection arose, until all the bacteria were killed or the HA hydrogel was completely degraded by the HAase enzyme. When the same system was preloaded with the platelet-derived growth factor BB and was applied on a subcutaneously *S. aureus*-infected rodent model, wound healing was promoted by the growth of blood vessels and new skin was formed, with no inflammation, within a 10-day period.

### 5.4. Redox-Responsive Polysaccharide Hydrogels

Chronic wounds, such as non-self-healing ulcers, exhibit excessive oxidant stress, which leads to upregulated ROS, reduced concentration of antioxidants, and a disturbed redox potential in the cells [86]. Glutathione (GSH) is a tripeptide that naturally occurs in the majority of mammalian cells and plays a significant role in biological processes, acting as an antioxidant agent. Its intracellular concentration varies depending on the pathological state, and is normally 0.5 mM for cells in a healthy state, compared to 10 mM for cancerous cells [87]. Furthermore, GSH levels have been found to be lower in diabetic foot ulcers. An interesting polysaccharide composite was developed by Gao et al. that can act simultaneously as a wound detection and point-of-care wound repair system, being sensitive to the variations in GSH concentration encountered in the wound environment. The hydrogel was fabricated using 2% HA and aminoethyl disulfide (AED), which acted as a dynamic covalent cross-linker triggered by the presence of GSH. The HA based-AED hydrogel presented an inherent redox response due to the AED-GSH conjugation, which resulted in the cleavage of the cross-linker and an increase in the swelling of the hydrogel measured as an increase in diameter of 63%. When tested in vitro, the hydrogel also aided fibroblast growth and proliferation over 7 days, presenting great promise for use as a scaffold to support fibroblast growth and wound repair [7].

Another ROS-responsive injectable hydrogel was developed for wound dressing applications. The hydrogel incorporated zeolite imidazole framework-8 (ZIF-8) nanoparticles within a sodium alginate and pectin network, cross-linked with calcium chloride. The ZIF-8 nanoparticles were coated with the 10-aminoacid neuro peptide (substance P, SP), which has been reported to accelerate wound healing and regulate inflammation through the induction of the mobilization of CD29(+) stromal cells into the circulation. Additionally, they were coated with poly(ethylene glycol)-PEG-thioketal (PEG-TK) to obtain a ROS-responsive feature. The SP@ZIF-8-PEG-TK@CA hydrogel was proposed as an excellent candidate for clinical studies, since its in vivo application in a full thickness excision mouse model promoted an early stage inflammatory response and high expression levels of CD206 at the later stage of the wound healing process, leading to high healing efficacy up to day 15 [88].

**Figure 10:**
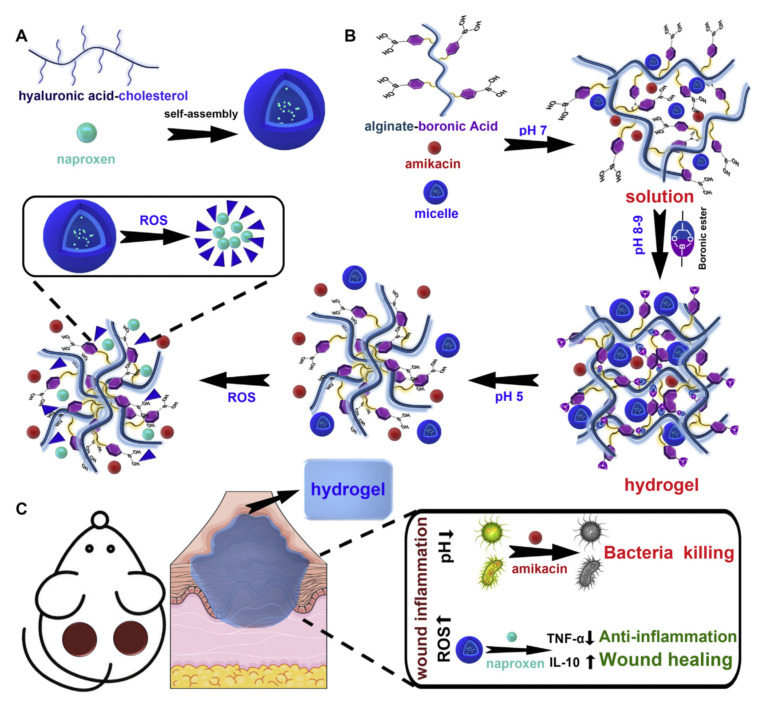
Schematic representation of (**A**) the self-assembly process (**B**) and the hydrogel formation. (**C**) Bactericidal and wound healing mechanisms in in vivo applications. Adapted with permission from [57].

The design of “smart” dual-responsive injectable hydrogels was reported by Hu et al. [57] for applications requiring on-demand antimicrobial activity and accelerated wound healing. The hydrogels responded to the acidic pH and increased ROS concentration encountered in the microbially infected wound areas [89,90]. The hydrogel was prepared by the interaction of ionized benzene boric acid (BA) with the diols of sodium alginate (ALG) that form reversible covalent bonds. Simultaneously, micelles encapsulating the anti-inflammatory drug naproxen were formed by the grafting of cholesterol (CHOL) onto hyaluronic acid (HA). Both the HA-CHOL micelles and the antimicrobial drug amikacin were encapsulated within the ALG-BA hydrogel, which presented dynamic properties such as stimuli-response, biocompatibility, self-healing properties, injectability, and the delivery of two anti-inflammatory and antibiotic drugs. Application on a bacterial-infected wound area induced the rapid disintegration of the hydrogel, due to the low pH in the infected area, and the release of the amikacin drug to kill the bacteria. Meanwhile, the increased ROS production in the area led to the degradation of HA [91], the dissociation of the HA-CHOL micelles, and the release of naproxen, which inhibited the inflammatory response of the macrophages and accelerated wound healing. In vitro results verified the effective antimicrobial action of the hydrogel and showed that the populations of *S. aureus* and *P. aeruginosa* were decreased by 90 and 98%, respectively. Moreover, the multifunctional hydrogel activity was evaluated in vitro by the cell scratch method, which showed the reduction of the concentration of the pro-inflammatory cytokine TNF-α by 2.80 times and the increase of the levels of the anti-inflammatory cytokine IL-10 by 2.41 times, compared to a control hydrogel that did not contain the drugs. Finally, the hydrogel was used as a wound dressing in a *P. aeruginosa*-infected rat model, and exhibited complete wound healing after 14 days of treatment (Figure 10).

### 5.5. Photo-Responsive Polysaccharide Hydrogels

Light responsive materials have recently emerged as a promising strategy for the treatment of bacterial infections during the wound repair process. Near infrared (NIR) light used as a biomedical tool exhibits superior properties, such as deep tissue penetration, low phototoxicity, and is a non-invasive treatment. In one approach, the conversion of light into heat can result in bacterial lysis in the targeted area. Yang et al. used tungsten disulphide nanosheets (WS2-NS2) triggered by NIR light to induce the heat accelerated release of the antimicrobial drug ciprofloxacin. A multifunctional nanocomposite dressing comprising dodecyl-modified chitosan and ciprofloxacin-loaded WS2-NS2 nanosheets was prepared by Schiff base formation. Chitosan provided a homogeneous porous structure with a pore diameter of 20 μm. Upon NIR light irradiation, photothermally generated hyperpyrexia triggered the bacterial dissociation as well as the on-demand release of the antimicrobial drug in the area. The irradiated nanocomposite completely inhibited *S. aureus* bacteria proliferation in vitro, and promoted an effective wound recovery in vivo, reaching 95% wound closure. Furthermore, the material exhibited self-healing, hemostatic, antioxidant, adhesion, and injectability properties [92].

A “smart” photo-responsive hydrogel dressing was developed by Zhao et al. [54] for accelerated wound healing. A supramolecular hydrogel was fabricated by the reversible host–guest interactions between azobenzene and β-CD grafted onto hyaluronic acid chains. The flexibility of the hydrogel was based on the photo-isomerization of azobenzene, which also has an affinity for the hydrophobic cavity of β-CD. Upon UV light irradiation, the bond between azobenzene and β-CD is distorted, leading to a partial dissociation of the hydrogel bonds. In the absence of UV light, the bond is reformed and the hydrogel structure is rearranged. Following a similar mechanism, the controlled release of the encapsulated EGF can be achieved on demand at the area of the wound. HA, one of the main components of the extracellular matrix, is the building block of the hydrogel, offering excellent biocompatibility [93]. Encapsulation of EGF in the HA hydrogel and manipulation of its release at the wound area ensures the stability of EGF and overcomes its rapid degradation by proteases found in the wound fluids [94]. The hydrogel exhibited superior wound healing efficiency when applied to a full-thickness skin defect mouse model and accelerated wound healing, while improving angiogenesis and granulation tissue formation after 10 days of treatment. A summary of all the stimuli-responsive polysaccharide composites described in this review is shown below in Table 2.

## 6. Conclusions and Future Perspectives

Polysaccharide hydrogels are attractive materials for wound healing applications, due to their biocompatibility, inherent water adsorption properties, biodegradability, and ability to functionalize them, giving interesting responsive properties to their structure. Polysaccharides have been commonly combined with other organic or inorganic materials to confer additional properties, such as higher mechanical properties, stimuli-response, injectability, and others. The development of stimuli-responsive hydrogels and their composites for wound healing applications has attracted great attention recently, with the most common examples including pH- and ROS-responsive hydrogels, as well as temperature-, enzyme-, and photo-responsive materials. Even though various chemical, biochemical, and physical stimuli have been extensively studied for inducing physicochemical, mechanical, and morphological changes in polysaccharide-based hydrogels, intense interest has sparked towards the direction of polysaccharide composite hydrogels that can respond to external stimuli such as light irradiation, ultrasounds, magnetic, or electric fields. Materials that alter their properties upon the application of an external stimulus can be very promising candidates for developing of next-generation wound dressings since the stimulus can be applied with great spatiotemporal control and thus remotely manage the degradation rate of the dressing as well as the release profile of the bioactive substances at the wounded area. Apart from this, externally triggered changes on a dressing could span its lifetime. For instance, light irradiation is a powerful external trigger that offers all the aforementioned advantages; however, studies of photo-sensitive polysaccharide hydrogels are limited in the literature. Considering the advantages of light-sensitive dressing, this could be an interesting and promising class of smart dressings for clinical use.

Injectable and self-healing polysaccharide hydrogels could be a very favorable solution and are also gaining popularity as effective wound dressings due to the advantages offered by these properties. When an injectable polysaccharide is combined with responsiveness to an internal stimulus such as temperature or pH, it has the advantage of more easily reaching the affected tissue when in a solution state, or transiting to a gel state when triggered by the body temperature, offering a hemostatic effect and in addition acting as a scaffold for epithelial cell proliferation during tissue regeneration at the last stage of wound healing. Simultaneously, a stimuli-responsive trait enables the release of the hydrogel’s drug cargo at a specific and targeted area when triggered by the pH conditions that are dominant in a microbial contaminated environment. Self-healing of a hydrogel is also advantageous in that case, since the spontaneous formation of new bonds occurring when old bonds are broken within a material can render the material stable throughout the healing process, especially when applied at wound areas that encounter a lot of strain. It has been previously demonstrated that injectable and self-healing hydrogels could improve the wound healing process compared to the use of gauze.

Although the design and synthesis of such “smart” materials has made good progress, there are still important challenges to address in the field. For example, understanding the materials’ function in the complex environment in vivo and programming their properties to match those of biological systems that can undergo dynamic changes in their properties and functions over time is still an ambitious goal. Moreover, addressing complicated chronic wounds, such as non-self-healing diabetic ulcers, remains an unmet need. The recent synthetic developments in multifunctional polysaccharide wound dressings strengthen the hope of combating acute and chronic wound diseases. Compared to the conventional wound dressing methods, these polysaccharide composite materials can be stimulated on site and offer a targeted response to the afflicted area, promoting wound healing, skin reconstruction, and additionally offering antimicrobial protection throughout the healing process. Collectively, stimuli-responsive polysaccharide hydrogels have the potential of combining all the aforementioned properties and responses depending on the area of application, type of wound, and conditions, therefore offering a “smart” and cost-effective solution for the development of wound healing dressings.

## Figures and Tables

**Table 1:** Major advantages and disadvantages of basic natural polysaccharides used in stimuli responsive wound healing applications.

Natural Polysaccharides	Advantages	Disadvantages
Chitosan/chitin	Facile modification methods, Versatility of functionalization, Inherent antibacterial activity, low cost	Performances of different batches greatly vary Non-soluble at neutral conditions Non-FDA approved Weak mechanical properties
Hyaluronic acid	Biocompatible, biodegradable, FDA-approved Inherent antioxidative properties Facile modification methods Versatility of functionalization High water-holding capacity	Weak mechanical properties Poor tissue-adhesion Fast degradation rates Expensive
Dextran	Biocompatible, biodegradable, FDA-approved Resistance to protein adsorption Facile modification methods Versatility of functionalization High water holding/absorbing capacity Low cost	Weak mechanical properties Poor tissue adhesion
Sodium alginate	Biocompatible, water-soluble Facile modification methods Low cost	Weak mechanical properties
Cellulose	Good biocompatibility, non-toxic, good mechanical properties	Solubility issues Non-adherent

**Table 2:** Summary of the stimuli-responsive polysaccharide composites described in this review.

Natural Polysaccharide	Composite/Active Agent	Synthetic Pathway/ Crosslinking	Stimuli- Responsiveness	Wound Healing Application	Clinical and Non-Clinical Application	Reference
Chitosan	Carbon dots	Solvent casting method	pH-monitoring	Antibacterial	In vivo (rats)	[70]
Quaternized chitosan and dopamine-oxidized dextran	Silver NPs and deferoxamine	Imine bonds and catechol-catechol adducts	pH-responsive	Antibacterial Angiogenic	In vivo (rats)	[53]
Oxidized hyaluronic acid and dihydrazide hyaluronic acid	Sisomicin sulfate or quaternized chitosan	Imine and acylhydrazonebonds	pH and enzyme-responsive	Antimicrobial	In vivo (mice)	[51]
Dopamine-grafted oxidized carboxymethyl cellulose	Poly(vinyl alcohol)-borax, neomycin	Hydrogen, borate ester, and imine bonds	pH-responsive	Antibacterial antioxidant	In vitro	[73]
Heparin	Poloxamer, Keratinocyte growth factor, ε-polylysine	Electrostatic interactions	Thermo-responsive	Wound healing of endometrial injury	In vivo (rats)	[74]
Hydroxypropyl chitin	Tannic acidFe^3+^ ions	Hydrogen and coordination bonds	Thermo-responsive pH-responsive	Antibacterial	In vivo (mice)	[45]
Hyaluronic acid	Ethylenediaminetetraacetic acid (EDTA)−Fe^3+^ complexes Platelet-derived growth factor	Coordination interactions	Enzyme-responsive	Antibacterial cutaneous regeneration	In vivo (mice)	[85]
Hyaluronic acid	Glutathione, Aminoethyl disulfide	Amide bonds	Redox-responsive	Wound monitoringantioxidant fibroblast growth	In vitro	[7]
Sodium alginate pectin	ZIF-8 nanoparticles calcium chloride, neuro peptide (SP), PEG-thioketal	Ionic cross-linking	Redox responsive	Wound healing acceleration	In vivo (mice)	[88]
Hyaluronic acid-cholesterol and alginate-boronic acid	Naproxen, amikacin	Boronic ester	Redox and pH-responsive	Wound healing acceleration, antibacterial, anti- inflammation	In vivo (rats)	[57]
Chitosan	Tungsten disulfide nanosheets (WS2-NS2), ciprofloxacin	Imine bonds	Photo-responsive	Antimicrobial, hemostaticantioxidant	In vivo (mice)	[92]
Hyaluronic acid- β-cyclodextrin and hyaluronic acid-azobenze	Epidermal growth factor	Host–guest interactions	Photo-responsive	Angiogenesis, granulation tissue formation	In vivo (mice)	[54]
Quaternized chitosan, and oxidized hyaluronic acid	Epidermal growth factor PEDOT:PSS, berberine	Imine bonds	pH-responsive	Antibacterial, wound healing acceleration	In vivo (mice)	[32]
Quaternized chitosan	Pluronic^®^F127 (PF127-CHO), curcumin	Imine bonds	pH-responsive	Antibacterial, hemostatic	In vivo (mice)	[52]
N-narboxyethyl chitosan, oxidized hyaluronic acid	Adipic acid dihydrazide, insulin	Acylhydrazones and imine bonds	pH-responsive	Diabetic wound healing, Re-epithelization Angiogenesis,	In vivo (rats)	[38]
N-carboxyethyl chitosan and oxidized hyaluronic acid	Polyaniline, amoxicillin	Imine bonds	pH and electro-responsive	antibacterial, antioxidant, angiogenesis, collagen disposition	In vivo (mice)	[39]
Oxidized hyaluronic acid	Antimicrobial peptide KK(SLKL)_3_KK	Imine bonds	pH-responsive	Antibacterial	In vivo (mice)	[66]
Oxidized pullulan	Polyethyleneimine, Pluronic F127	Imine bonds	pH and thermo responsive	Antibacterial, hemostatic, stimulate angiogenesis, remodelling and re-epithelialization	In vivo (mice)	[56]

## Data Availability

The data presented in this study are available in the article.

## Abbreviations

Abbreviation	Name
ROS	reactive oxygen species
GSH	glutathione
CS	chitosan
QCS	quaternized chitosan
HBCS	hydroxybutyl chitosan
CMCS	carboxymethyl chitosan
GTMAC	glycidyl trimethyl ammonium chloride
OHA	oxidized hyaluronic acid
PEG	poly(ethylene glycol)
HPC	hydroxypropyl cellulose
OCAPS	octa (γ-chloroammoniumpropyl) silsesquioxane
rGO	reduced graphene oxide
TEOS	tetraethyl orthosilicate
OD	oxidized dextran
HTCC	N-(2-hydroxypropyl)-3-trimethylammonium chitosan chloride
HA-ADH	dihydrazide hyaluronic acid
BBH	berberine
EGF	epidermal growth factor
PEDOT: PSS	poly(3, 4-ethylenedioxythiophene):poly(styrene sulfonate)
PF127-CHO	aldehyde pluronic F127
OD-DA	oxidized dextran
DFO	deferoxamine
CECS	carboxyethyl chitosan
ADH	adipic acid dihydrazide
AEDa	Aminoethyl disulfide
β-CD	β-cyclodextrin
HA-CD	HA conjugated with β-cyclodextrin
HA-Azo	HA conjugated with trans-azobenzene
APu	aldehyde pullulan
PEI	polyethylenimine
ADSC	adipose mesenchymal stem cell
HPCH	hydroxypropyl chitin
TA	tannic acid
ALG-BA	alginate backbone
CHOL	cholesterol
CP	chitosan-*g*-polyaniline
RGD	arginine-glycine-aspartate
CM-TM-CS	carboxymethyl-trimethyl chitosan
GelMA	gelatin methacrylamide
MMPs	matrix metalloproteinases
HIF-1α	factor-1 alpha
VEGF	vascular endothelial growth factor
SS	sisomicin sulfate
AgNPs	silver nanoparticles
PB	poly(vinyl alcohol)-borax
OCMC-DA	dopamine grafted oxidized carboxymethyl cellulose
EPL	ε-poly-L-lysine
KGF	keratinocyte growth factor
HP	heparin-modified poloxamer
FEP	fluorinated ethylene propylene
EDTA	ethylenediaminetetraacetic acid
ZIF-8	zeolite imidazole framework-8
PEG-TK	PEG-thioketal
BA	boric acid
HA	hyaluronic acid
NIR	Near infrared
WS2-NS2	tungsten disulphide nanosheets
